# Occupational asthma following single exposure to polyurethane foam containing methylene diphenyl diisocyanate – A case report

**DOI:** 10.1016/j.toxrep.2025.102150

**Published:** 2025-10-28

**Authors:** Albin Stjernbrandt

**Affiliations:** Department of Epidemiology and Global Health, Umeå University, Umeå 901 87, Sweden

**Keywords:** Asthma, Occupational, Isocyanates, Reactive airways dysfunction syndrome

## Abstract

Diisocyanates are a group of chemicals used in many different applications, such as plastics, foams, coatings, adhesives, and sealants. Prolonged occupational exposure can result in severe asthma. This case report presents a non-smoking male without any previous respiratory disease, where severe obstructive airway symptoms developed during a single event with high airborne exposure to polyurethane foam containing methylene diphenyl diisocyanate during the coating of a large vehicle. The subject was subsequently diagnosed with occupational asthma based on a significant variability in a two-week peak expiratory flow curve and a positive metacholine challenge. Despite aborted exposure and optimized asthma treatment, the subject continued to experience debilitating airway symptoms. This case report demonstrates that severe asthma can develop following a single exposure to polyurethane foam containing methylene diphenyl diisocyanate, underscoring the importance of preventive measures in workplaces where such chemicals are used.

## Highlights

• Occupational exposure to diisocyanates can lead to asthma development.
• One-time exposure without previous sensitization can be enough to cause asthma.
• The diagnosis of diisocyanate asthma should ideally be based on a provocation test.

## Graphical Abstract

**Figure:**
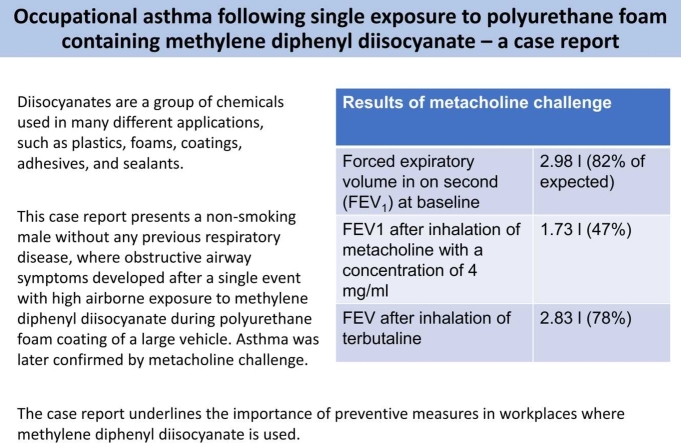

## 1 Introduction

Diisocyanates are highly reactive, low-molecular-weight chemicals that are used for cross-linking polymer chains in many different applications, such as plastics, foams, coatings, adhesives, and sealants. Although they have many beneficial chemical characteristics in industrial applications, diisocyanates are also well-known to cause occupational asthma [1]. In fact, a recent review concluded that exposure to diisocyanates is a leading cause of occupational asthma in the European Union [2]. The common pathomechanistic understanding is that a worker is first immunologically sensitized to a specific diisocyanate compound by airborne or dermal exposure, and then reacts to low-level airborne exposure with airway obstruction and inflammation [2]. This mechanism usually requires prolonged exposure for sensitization. However, an alternative, non-immunologic pathway has been suggested, where the acute irritative effects of high exposure can result in immediate bronchopulmonary injury and subsequent obstructive airway symptoms, sometimes referred to as reactive airway dysfunction syndrome (RADS) [3]. However, RADS mandates exposure to very high concentrations of irritant vapors, gases, or fumes and is often described in conjunction with industrial accidents. Common agents for RADS include chlorine, nitrogen oxides, acetic acid, and sulfur dioxide, although toluene diisocyanate (TDI) has also been suggested [3].

Methylene diphenyl diisocyanate (MDI) is the most common diisocyanate globally and is often used in polyurethane (PU)-based products such as foam insulation [4]. Since the volatility of MDI is lower than that of other common diisocyanates such as TDI and hexamethylene diisocyanate (HDI), it has been proposed as a safer alternative [4]. Much of the previous research has focused on TDI, even though new uses for MDI, such as spray-on PU foam insulation, have resulted in new opportunities for exposure [4]. There is a lack of knowledge regarding exposure-effect relationships, i.e., what concentration and duration of exposure are required to cause obstructive airway disease [1]. Also, to the author’s knowledge, there is no previous description of the sudden onset of asthma or RADS after a single exposure to MDI. This case report illustrates the immediate development of severe asthma following a single event with exposure to PU foam containing MDI.

## 2 Case presentation

The patient was a white, non-smoking male in his 60 s without any previous respiratory disease. There were no airway diseases reported among first-degree relatives. He had a desk job and no previous occupational or leisure-time exposure to vapors, gases, dusts, or fumes. As part of his work, he was visiting a factory in another country specializing in PU foam spraying on the chassis of heavy vehicles for thermal and sound insulation. A two-component PU foam containing methylene diphenyl diisocyanate was used, which was stored in closed barrels and mixed within an air-pressured spray gun. He was invited to inspect the process close-hand, where city buses were lifted approximately two meters off the ground and closed off from the rest of the factory by a plastic protective film running from the sides of the vehicle to the floor to limit overspray. The area beneath the bus had no local exhaust ventilation. The patient was using a simple filtering half mask and was standing in this confined space for somewhere between 1,5–2 h while a worker wearing full protective clothing and a supplied-air respirator sprayed the PU foam. Within this period, the patient experienced increasing trouble breathing, a dry cough, and nausea, forcing him to leave the factory prematurely. Because of continuing severe airway symptoms, he was subsequently transported to the local emergency department.

At the hospital, clinical examination revealed a man of normal stature with no peripheral edema or jugular vein stasis, but with a persistent cough and difficulty breathing. Auscultation of the lungs revealed sibilant rhonchi, while the heart sounds were unremarkable. The oxygen saturation was 99 %, the respiratory rate 16/min, blood pressure 148/80 mmHg, and pulse 70 bpm. A standing chest X-ray revealed normal findings. Extensive blood sampling, including full blood count, erythrocyte sedimentation rate, C-reactive protein, and D-dimer, showed normal findings. PCR for chlamydia pneumoniae, mycoplasma pneumoniae, enterovirus, influenza A and B virus, parainfluenza virus, metapneumovirus, rhinovirus, and respiratory syncytial virus were all negative. He was treated with a high dose of inhaled budesonide (4 g) and discharged with a prescription of budesonide 800 µg daily and terbutaline 0,5 mg on demand.

A two-week peak expiratory flow (PEF) curve was started after the initial incident, with values ranging between 440 and 610 L/min and several accounts of significant daily variation (>20 %). Spirometry and a metacholine challenge were performed 50 days after the initial incident, and the forced expiratory volume in one second (FEV_1_) decreased from 2.98 L (82 % of expected) to 1,73 L (47 %) after inhalation of metacholine with a concentration of 4 mg/ml. After a subsequent 1.0 mg inhalation of terbutaline, FEV_1_ increased again to 2.83 L (78 %). The provocative concentration of metacholine for a 20 % decrease in FEV_1_ (PC_20_) was calculated to be 2.7 mg/ml, indicating mild bronchial hyper-responsiveness [5]. The forced vital capacity (FVC) was 3.1 L (66 %) and slow vital capacity (SVC) 3.0 L (63 %). A full lung function test was also performed and showed an increased FVC of 3.5 L (75 %), normalized SVC of 4.2 L (91 %), inspiratory capacity of 3.3 L (86 %), total lung capacity of 7.0 L (88 %), and diffusion capacity of carbon monoxide (DLCO) of 74 % that was considered slightly impaired. However, both the FVC and DLCO were deemed unreliable because of prolonged coughing. Sampling of fractional exhaled nitric oxide was 19 ppb (reference value <25 ppb for adults). Blood sampling for specific IgE antibodies for diisocyanates (MDI, HDI, and TDI) was negative, and the total IgE level was not elevated (15.7 kU/l). High-resolution computed tomography of the chest showed no pathological changes in the lung parenchyma, no pleural effusion, and no enlarged lymph nodes. Since sufficient symptom relief was not obtained on the current treatment regimen, budesonide was increased to 1600 µg daily. A long-acting β-agonist was also added, in the form of formoterol 18 µg daily. Despite the augmented treatment, he continued to experience symptoms and rated 10 points on the asthma control test, indicating inadequate asthma control [6]. He reported a continuous dry cough that was exacerbated by cold air and effort. He also experienced rhinitis, hoarseness, and weakness of voice. He had to stop to catch his breath after walking less than two flights of stairs. These symptoms persisted at follow-up six months after the incident. The patient was assessed at the Occupational and Environmental Health Clinic at the University Hospital of Umeå, and it was concluded that he had developed occupational asthma due to exposure to MDI. The patient was subsequently referred to a pulmonologist for further optimization of the treatment.

## 3 Discussion

This case report illustrates the immediate onset of obstructive airway symptoms following a single event with high exposure to PU foam containing MDI. No exposure measurements were conducted at this instance, but several previous studies have documented exposure levels of MDI in similar spray-on applications. A U.S. study investigated companies (N = 13) applying truck bed linings containing MDI using handheld spray guns in closed-off areas [7]. The mean airborne MDI concentration (measured during 10–20 min) ranged from 0.05 to 6.50 mg/m^3^, and in seven of the 13 companies, the Occupational Safety and Health Administration (OSHA) ceiling limit of 0.20 mg/m^3^ was exceeded. Moreover, several workers employed at these companies had filed workers’ compensation claims for new-onset asthma. The National Institute for Occupational Safety and Health also conducted MDI exposure assessments based on measurements from similar spray-on applications of MDI in confined spaces (N = 6) [8]. The mean airborne MDI concentration (measured during 16–43 min) was 0.99 mg/m^3^ for processes with low temperature and pressure, and 0.78 mg/m^3^ for processes with high temperature and pressure. Thus, in both settings, the OSHA ceiling limit was exceeded several times, and the patient in the current case description was likely exposed to somewhat similar concentrations. These levels are also much higher than what has been shown in other industrial processes, such as 0.03–3.3 µg/m^3^ in Finnish PU factories [9] and 0.04–7.8 µg/m^3^ in Swedish PU industries [10]. To combat the ongoing issue of occupational diisocyanate asthma, the Swedish limit value for diisocyanates is currently being lowered to 0.006 mg/m^3^ [11].

Diagnosing occupational asthma is a complex and multi-step procedure [12]. The starting point is work-related asthma symptoms, which should ideally be investigated by assessment of non-specific bronchial hyperresponsiveness (e.g., mannitol or metacholine challenge) as well as immunological sensitization (e.g., skin prick test or specific IgE antibodies). If there is bronchial hyperresponsiveness but no clear indication of immunological sensitization, the next step is to perform serial PEF measurements or conduct a specific inhalation challenge (using the suspected substance), which both may confirm the diagnosis [12]. A specific inhalation challenge can be considered the most reliable diagnostic tool, although it is not always available or safe to perform.

According to the literature, symptoms of diisocyanate asthma commonly begin insidiously after an extended latency period of several months to years after starting exposure [1]. There are several case reports of occupational asthma in relation to MDI. However, these describe recurrent exposure rather than single events. For instance, Baur et al. reported on a non-atopic worker who occasionally sprayed PU foam containing MDI for use as a packing material and subsequently developed asthmatic symptoms. Specific inhalation challenge with MDI resulted in immediate bronchial obstruction with reduced FEV_1_. Significant decreases in vital capacity and diffusion capacity were also seen. However, skin prick testing for MDI was negative, and there were no specific IgE antibodies [13]. Littorin et al. described a non-atopic but smoking worker without previous obstructive airway disease who repaired PU-containing conveyor belts, using heating or gluing, and therefore was recurrently exposed to MDI [14]. After an incident with high exposure while imprinting on a long conveyor belt for several hours, he developed acute respiratory distress and was subsequently diagnosed with new-onset asthma. The exposure scenario was experimentally repeated, resulting in an airborne concentration of MDI of 15 µg/m^3^ in the respiratory zone. Spirometry revealed moderate obstruction, but the diffusion capacity was normal. Skin prick testing of MDI was positive, and there were both IgE and IgG antibodies to MDI. Leroyer et al. described a smoking worker without any history of atopy or asthma who worked at a foundry making cores, which involved frequent exposure to MDI [15]. After an accidental spill of MDI-containing solvent, he experienced obstructive airway symptoms. Later investigation revealed that he had developed asthma, and a specific inhalation challenge with MDI showed a fall in FEV_1_ of 22 % after the end of exposure. Metacholine challenge was also positive with a PC_20_ of 3 mg/ml. Zeiss et al. described a worker repeatedly exposed to MDI while coating pipes with PU foam, who developed occupational asthma [16]. Blood sampling revealed both IgE and IgG antibodies for MDI, and the authors concluded that the asthma was likely mediated by type 1 allergy. Finally, Wisnenewski et al. reported on a male worker in an aluminum foundry who had been recurrently exposed to an MDI-containing resin when making molds for casting. He was diagnosed with occupational asthma and eventually died from an asthma exacerbation at work. Post-mortem analysis revealed IgE and IgG antibodies to MDI, and lung tissue sections obtained during autopsy were used for immunochemical detection of MDI in situ. These cases illustrate how recurrent exposure to MDI can lead to asthma development. There are also case reports where asthma has developed after a single exposure to diisocyanates, but none of these involve exposure to MDI. For instance, Luo et al. reported on two police officers who responded to a traffic incident where a tank car with TDI had been overturned and was leaking. Both subjects reacted with obstructive airway symptoms immediately after exposure and were assessed by an occupational physician to have chronic asthma-like illness after brief but likely high exposure [17]. In a case report by Boulet, a worker spraying TDI-containing PU foam reported asthmatic symptoms after one-time exposure to high concentrations. Testing revealed mild bronchial hyperresponsiveness to metacholine (PC_20_ 4,3 mg/ml). Subjected to TDI in a provocation chamber, immediate symptoms recurred, FEV_1_ fell by 26 %, and complete recovery was achieved within two hours following administration of albuterol and beclomethasone. These case reports show the potential for asthma-like disease after single exposure events, but with TDI as the likely culprit.

The current case report had a somewhat similar clinical presentation but involved MDI instead of TDI. Since the patient reacted immediately at first exposure to MDI, had no atopic predisposition, and lacked diisocyanate-specific IgE antibodies, it is plausible that his asthma was irritant-induced rather than mediated by type 1 allergy. However, since the treatment was not effective during follow-up, other potential triggering factors should be considered. Despite our best efforts, the investigation did not reveal any other environmental or personal health factors of relevance for the development of asthma in this patient. The diagnostic value of specific IgE antibodies for diisocyanates has previously been thoroughly investigated. For instance, Baur et al. collected blood tests from diisocyanate-exposed subjects who reported work-related symptoms (predominantly airway symptoms; n = 1095) and found that only about 14 % presented with specific IgE antibodies for diisocyanates [18]. More recent studies have defended the diagnostic value of specific IgE antibodies, concluding on a low sensitivity but high specificity for diisocyanate asthma [19]. There is ongoing work to determine suitable biomarkers for predicting diisocyanate-induced occupational asthma [20]. However, a specific inhalation challenge using increasing concentrations of the specific diisocyanate is currently the most convincing diagnostic approach, but it is technically challenging and not readily available at all occupational medicine clinics. Previous claims that MDI should be considered less hazardous than TDI and HDI because of less volatility can be partly refuted by the fact that MDI appears to be more asthmagenic [21].

This case report has several limitations. No exposure measurements could be conducted since the factory was in another country, and this is an obvious limitation in inferring causality. Moreover, there were no lung function tests performed before the onset of symptoms, and no previous medical investigation regarding allergies, which means that previous airway disease or allergy cannot be entirely ruled out. Finally, a specific inhalation challenge could not be conducted. Strengths include other thorough medical investigation at an occupational medicine clinic and six months of follow-up after the incident.

This case report highlights the importance of preventive measures in workplaces where MDI is used, and detailed guidance on preventing exposure to diisocyanates has previously been outlined [22]. Measures should include thorough risk assessments, efforts to replace MDI with less hazardous substances, the use of less reactive prepolymers, limiting exposure at the source through confinement and local exhaust ventilation, tailoring personal protective equipment (including supplied-air full-facepiece respirators), and recurrent medical surveillance of exposed workers. There is also a need to advance the knowledge on the pathophysiological mechanisms of immediate-onset asthma after diisocyanate exposure.

## CRediT authorship contribution statement

**Albin Stjernbrandt:** Writing – original draft, Methodology, Investigation, Data curation, Conceptualization.

## Informed consent

The patient has provided informed consent for this case report to be written.

## Funding

The author did not receive any specific funding, but the research was performed as part of employment at 10.13039/501100004885Umeå University.

## Declaration of Competing Interest

The authors declare that they have no known competing financial interests or personal relationships that could have appeared to influence the work reported in this paper.

## Data Availability

The data supporting this case report are not publicly available due to ethical restrictions.
